# 

**Published:** 1905-05-06

**Authors:** 


**NOTICE TO CORRESPONDENTS.**

All MSS., Letters, Books for Review, and other matters intended for the Editor should be addressed THE EDITOR, "THE HOSPITAL," THE HOSPITAL BUILDINGS, 28 & 29 SOUTHAMPTON STREET, STRAND, W.C.

The Editor cannot undertake to return rejected MS., even when accompanied by stamped directed envelope.

**Notice to Advertisers and Subscribers.**

All Advertisements, Orders for Copies of THE HOSPITAL, and Business Communications should be addressed to **THE MANAGER (Not to the Editor),** THE HOSPITAL, 28 & 29 Southampton Street, Strand, London, W.C.

The Cost of Subscription, post free, is at follows:—Per week, 3½d.; per quarter, 3s. 3d.; per half-year, 6s. 6d.; per annum, 13s. Foreign and Colonial:—Per annum, 19s. 6d., payable, in all cases, in advance.

**NOTICE.—Vol. XXXVII. of "The Hospital" October 1904 to March 1905), in handsome cloth binding, will shortly be on sale at the office of this Journal, price 10s. 6d. Cases for binding the Numbers contained in this Volume 2s. Index to Vol. XXXVII., 3½d., post free.**

**The Monthly Part for May is now ready. Price 1s., by post, 1s. 3d.**

**A COMPLETE HANDBOOK OF MIDWIFERY.**
By J. K. WATSON, M.D. **6s.** net, **6s. 4d.** post free.
Illustrated with 143 Diagrams. Cloth gilt.

**PRACTICAL GUIDE TO SURGICAL BANDAGING AND DRESSINGS.**
By WM. JOHNSON SMITH, F.R.C.S. **2s.** post free.
Suitable size for the apron pocket.
Profusely Illustrated.

**SURGICAL WARD-WORK AND NURSING.**
By ALEXANDER MILES, M.D. Edin. **3s. 6d.** net, **3s. 10d.** post free.
Second Edition. Illustrated. Cloth gilt.

**THE EXAMINATION OF THE URINE.**
By J. K. WATSON, M.D. **1s.** net, **1s. 1d.** post free.
Cloth boards. Suitable size for the apron pocket.

**THE NURSES' PRONOUNCING DICTIONARY OF MEDICAL TERMS AND NURSING TREATMENT.**
Compiled by HONNOR MORTEN. **2s.** post free.
Fifth Edition, with Phonetic Pronunciation, Revised and Edited by Mary I. Burdett.
Suitable size for the apron Pocket. Cloth.

**THE BURDETT SERIES OF NURSING TEXT-BOOKS. All Subjects.**
**1s.** post free.

**CASE-BOOKS**
**For all Branches of Nursing.**

**NURSING: ITS THEORY AND PRACTICE.**
By PERCY LEWIS, M.D. **3s. 6d.** post free.
Illustrated. Cloth gilt.

**ELEMENTARY ANATOMY AND SURGERY FOR NURSES.**
By WM. McADAM ECCLES, M.S., M.B.
**2s. 6d.** post free.
Profusely Illustrated. Cloth.

**ELEMENTARY PHYSIOLOGY FOR NURSES.**
By C. F. MARSHALL, M.D., B.Sc., F.R.C.S.
**2s.** post free.
Profusely Illustrated. Cloth.

**GYNÆCOLOGICAL NURSING.**

By G. A. HAWKINS-AMBLER, F.R.C.S.

Cloth, **1s.** post free.

**FEVERS AND INFECTIOUS DISEASES.**
By WILLIAM HARDING, M.D.
Cloth, **1s.** post free.

**NEW CASE-BOOK—PRIVATE NURSING NIGHT AND DAY.**
Large size, **3d.** net; **4½d.** post free.

**CHARTS**
**and every Requirement for Nursing Treatment.**

### "The NURSING MIRROR"
### "The Nurse's Own Paper."
**Price One Penny weekly. Of all Booksellers and Newsagents.**

NEW CATALOGUE OF NURSING MANUALS POST FREE ON APPLICATION.

*The above Works can be obtained of all Booksellers, or of*

**The Scientific Press, Ltd.,** 28 & 29 SOUTHAMPTON STREET, STRAND, LONDON, W.C.

